# Exploring Factors Contributing to Effective Teaching in Dental Clinical Settings: Perceptions of Dental Students

**DOI:** 10.3390/dj13020075

**Published:** 2025-02-08

**Authors:** Rayan Sharka, Mansour Alghamdi, Ammar Almarghlani, Hassan Abed, Shahad Alluqmani, Ruza Alhazmi, Jameel Abuljadayel

**Affiliations:** 1 Department of Oral and Maxillofacial Surgery, Faculty of Dental Medicine, Umm Al-Qura University, Makkah 21955, Saudi Arabia; mhghamdi@uqu.edu.sa; 2 Department of Periodontics, Faculty of Dentistry, King Abdulaziz University, Jeddah 21589, Saudi Arabia; mabdulhalim@kau.edu.sa; 3 Basic and Clinical Oral Sciences, Faculty of Dental Medicine, Umm Al-Qura University, Makkah 21955, Saudi Arabia; hhabed@uqu.edu.sa; 4 Dental Teaching Hospital, Faculty of Dental Medicine, Umm Al-Qura University, Makkah 21955, Saudi Arabia; s442002667@uqu.edu.sa (S.A.); s442011043@uqu.edu.sa (R.A.); 5 Department of Preventive Dentistry, Faculty of Dental Medicine, Umm Al-Qura University, Makkah 21955, Saudi Arabia; jaabuljadayel@uqu.edu.sa

**Keywords:** dentistry, clinical clerkship, dental education, clinical teacher, teaching

## Abstract

**Background:** Clinical teaching is crucial to dental education as it shapes the identity and professional development of students. However, there is a lack of research regarding the responsibilities of clinical teachers, their professional behavior, pedagogical approaches, and their influence on students’ clinical learning outcomes. This study aims to identify factors that students perceive in clinical teachers and how these factors influence the effectiveness of their clinical education. **Methods:** This cross-sectional study involved predoctoral and graduate dental students (N = 354). Data were collected using a questionnaire adapted from the Stanford Faculty Development Program (SFDP) scale and open-ended questions. Simple and multiple linear regression analyses were conducted to examine associations and predictive capabilities of the SFDP constructs for clinical teaching effectiveness. Data were analyzed using SPSS version 29. **Results:** A total of 332 responses were received, with a 94% response rate. Significant positive correlations were found between SFDP constructs and clinical teaching effectiveness. The evaluation construct had the strongest correlation (r = 0.480, *p* < 0.001), explaining 23.1% of the variance. The communication of clinical goals had the second strongest correlation (r = 0.415, *p* < 0.001), explaining 17.3%. The lowest correlation was for promotion of understanding and retention (r = 0.332, *p* < 0.001), explaining 11%. Cronbach’s alpha ranged from 0.607 to 0.783. **Conclusions:** This study highlighted key factors influencing the effectiveness of clinical teaching, including evaluation quality, clear communication of clinical goals, supportive learning environments, and effective feedback. Clinical teachers’ respect for students also facilitates successful education. More studies are required to explore additional factors across different contexts.

## 1. Introduction

Clinical teaching is fundamental to dental education since it shapes the identity and professional development of dental students [1]. It encourages students to gain practical experiences and refine important clinical skills in a safe environment under the guidance of experienced educators [2]. Such real-world experience is crucial to developing student’s confidence and competency in performing dental procedures [1]. Evidence indicates that the effectiveness of clinical teachers is pivotal in achieving the clinical outcomes of students [3,4].

Furthermore, effective clinical teaching emphasizes the importance of patient-centered care and professionalism [5], helping students to understand the nuances of patient care and clinical decision-making [6,7]. Close mentoring and constructive feedback allow students to continually enhance their hands-on skills and clinical judgment [8]. Ultimately, clinical teaching ensures that dental graduates are not only skilled in their technical abilities but also equipped to provide optimum patient care [7]. 

Effective clinical teaching is multidimensional with clinical teachers possessing a wide range of knowledge, skills, and personal attributes and knowing when and how to apply them [9]. This includes technical expertise and clinical proficiency as well as the ability to communicate effectively, demonstrate empathy, and provide constructive feedback. The integration of evidence-based teaching practices further enhances the learning experience, ensuring that students are exposed to the most current and effective clinical techniques [10].

Monitoring clinical teaching in dental programs is essential to ensure that students receive sufficient guidance and acquire the necessary skills to become proficient dentists [7]. These evaluations also ensure compliance with higher educational standards and contemporary professional and academic criteria [11]. In addition, most accreditation bodies require periodic reviews to ensure that the school meets the accepted standards of education [12]. Furthermore, such assessments provide valuable insights for clinical teachers, allowing them to refine their pedagogical methods to promote student learning [13].

The dental education literature predominantly focuses on the development of clinical curricula, course specifications, and educational materials, with a deficiency in research and discussion about the clinical teachers. A previous systematic review revealed that most studies concentrated on teaching methodologies, supportiveness, role modeling, and the provision of feedback but lacked a precise definition of what constitutes an effective clinical teacher. Consequently, the authors advocated for the development of more valid and comprehensive evaluation instruments to better assess clinical teaching [14].

Additionally, previous studies emphasized the need for more research focused on measuring undergraduate dental students’ perceptions of their clinical education to provide insights into the effectiveness of current teaching methods and highlight areas for improvement [13,15]. By exploring students’ views, clinical teachers can better tailor their approaches to meet their learners’ needs and expectations, ultimately enhancing the overall educational experience [13,15,16]. There is a lack of research regarding the responsibilities of clinical teachers, their professional behavior, pedagogical approaches, and their influence on student learning outcomes [11]. It is crucial to address this gap since the efficacy of clinical education is profoundly affected by the quality and competencies of the teachers who impart it [13,15]. Therefore, a more balanced approach that includes a thorough examination of clinical teachers is essential for advancing dental education. Also, research on undergraduate dental students’ perceptions of their clinical education is vital for advancing the field, enabling educators to refine their teaching methods, address students’ needs, and ultimately produce competent and confident dental professionals. Therefore, this study aimed to identify the key factors perceived by dental students as being important for effective clinical dental teaching.

### Theoretical Framework

The Stanford Faculty Development Program (SFDP) construct was employed as the theoretical framework [17]. This comprehensive framework, originally designed to enhance the teaching skills of medical educators, focused on the following seven key categories essential for effective clinical teaching [18]: (1) Learning climate: This construct focuses on creating a positive and supportive learning environment, including aspects such as respect and encouragement. In the clinical teaching context, it also involves establishing a safe environment for students to treat patients confidently and professionally. (2) Control of the session: This construct involves the clinical teacher’s ability to manage the session effectively, including time management, maintaining focus, and setting relevant guidelines for the clinical sessions. (3) Communication of clinical goals: This construct emphasizes clear communication of the clinical session’s goals and objectives, including clinical requirements and deadlines. (4) Promotion of understanding and retention: This construct deals with strategies to enhance students’ comprehension and retention of clinical procedures. It includes techniques such as summarizing key points, using examples, adopting an interactive approach, and encouraging active participation. (5) Evaluation: This construct involved assessing students’ understanding and ensuring they could apply knowledge to specific patients’ needs. It highlights the importance of a fair assessment of the clinical skills students developed throughout the sessions. (6) Feedback: This construct pertains to providing timely and specific feedback essential for student development. It focuses on how feedback is delivered and its impact on student improvement. (7) Self-directed learning: This construct encourages students to take responsibility for their own learning, fostering skills such as critical thinking, problem-solving, and independent study.

Despite its widespread application and demonstrated efficacy in medical education, the SFDP scale has not been effectively utilized within dental education. This gap presents an opportunity for dental education to leverage the SFDP scale. Therefore, this scale was applied in this study to measure dental students’ perceptions of their clinical teaching to guide faculty development and enhance clinical teaching practices [19,20].

**Figure 1:**
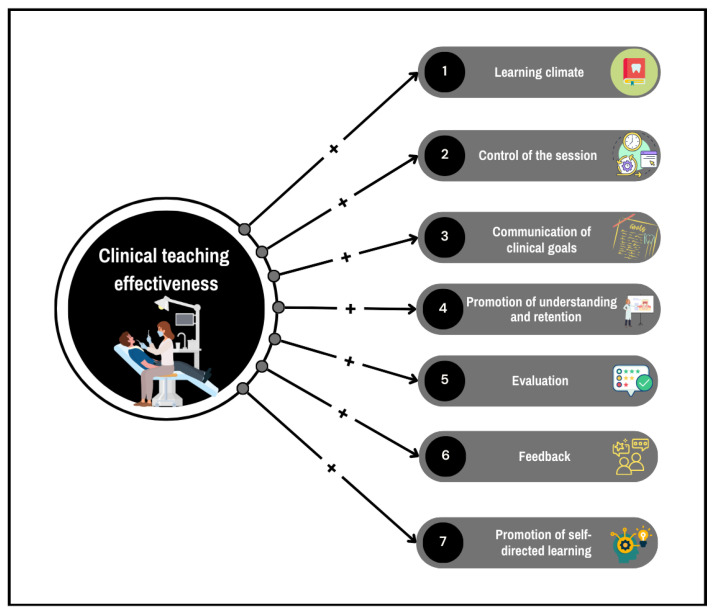
The proposed model.

The dependent variable in the proposed model was ‘clinical teaching effectiveness’, which measured students’ perceptions of the clinical teaching they received. This variable captures the direct impact of clinical teacher characteristics on students’ clinical learning experiences (Figure 1). Based on this model, this study aimed to test eight hypotheses:

**H1:**  Learning climate is positively associated with clinical teaching effectiveness.

**H2:**  Control of the session is positively associated with clinical teaching effectiveness.

**H3:**  Communication of clinical goals is positively associated with clinical teaching effectiveness.

**H4:**  Promotion of understanding and retention is positively associated with clinical teaching effectiveness.

**H5:**  Evaluation is positively associated with clinical teaching effectiveness.

**H6:**  Feedback is positively associated with clinical teaching effectiveness.

**H7:**  Promotion of self-directed learning is positively associated with clinical teaching effectiveness.

**H8:**  Overall, the proposed model significantly predicts clinical teaching effectiveness perceived by dental students.

The null hypothesis is that no SFDP factors are positively associated with clinical teaching effectiveness as perceived by dental students.

## 2. Materials and Methods

### 2.1. Ethical Considerations

The ethics and research committee at Umm Al-Qura University provided ethical approval for this study, with the reference number HAPO-02-K-012-2024-02-2060.

### 2.2. Study Design and Participants

This cross-sectional study was conducted at the dental teaching hospital within the Faculty of Dental Medicine at Umm Al-Qura University from 1 September to 31 October 2024. All predoctoral dental students in their clinical years (N = 299) and graduate dental students in their internship year (N = 55) enrolled in the 2024–2025 academic year were invited to take part in the study. A formal email from the Vice Deanship for Quality and Development, which included the participant information sheet, consent form, and the questionnaire link, was sent to the students’ university email addresses at the end of the first semester. Students could access the link exclusively through their university accounts. A reminder email was sent four weeks later. The Raosoft sample size calculator was utilized to determine the minimum sample size needed for the study. Based on a response distribution of 50%, a total participant pool of 354, a 95% confidence interval, and a 5% margin of error, the required sample size was calculated to be 185 participants.

### 2.3. Measures

The questionnaire was administered through a web-based survey platform (Microsoft Forms) provided to students in the email invitation. The first section included participant information and consent statements. The second section comprised three demographic questions pertaining to the respondent’s gender, age, and academic level. The third section included 23 items adapted from the SFDP scale used and validated in previous studies [18,20]. The clinical teaching effectiveness construct was measured using 3 items, such as “Generally, I received effective clinical teaching”. In line with previous research, responses were collected using a 5-point Likert scale, ranging from 1 (strongly disagree) to 5 (strongly agree) [18,20].

The developed questionnaire underwent a content validity process, which included a review by an expert panel of five clinical teachers with experience in scale development from various departments, including public health, restorative dentistry, periodontics, and prosthodontics, at the authors’ institution. These experts assessed the relevance and comprehensiveness of the items, ensuring clarity and conciseness. The main modification to the original instrument utilized by previous studies was changing the word “medical” to “dental”. Additionally, some items were reworded to suit the clinical teaching context rather than the classroom teaching context, such as “The teacher should use blackboard or other visual aids” to “The clinical teacher should use visual aids (i.e., photos, videos, and models) to explain the clinical procedure” and “The teacher should repeat the class goals periodically” to “The clinical teacher should state the deadline of performing clinical requirements periodically”.

The final part of the questionnaire included an optional open-ended question: “Please share the professional or general criteria you value in a clinical teacher that impact the effectiveness of your clinical teaching?” The aim of this question was to elicit detailed responses about students’ perceptions of the criteria they valued in clinical teaching, expressed in their own words. This question allowed students to freely express their thoughts, uncovering nuanced insights into the factors that contributed to effective clinical instruction. This qualitative approach complemented the quantitative data collected from the structured scale in the earlier sections of the questionnaire.

The final version of the questionnaire was piloted with ten graduate students not included in the main analysis to evaluate its clarity, consistency, validity, and the estimated time required to complete the questions. Feedback from the pilot study was used to make necessary adjustments. For example, one item was modified for clarity from “The clinical teacher should be attentive to the clinical session time” to “The clinical teacher should keep the clinical session within the scheduled timeframe”. Another item was modified from “The clinical teacher should avoid digressions during the clinical sessions” to “The clinical teacher should set clear guidelines for the clinical sessions”. This process helped refine the questionnaire, enhancing its overall quality and suitability for the main study.

### 2.4. Data Analysis

Descriptive statistics were used to calculate percentages and frequencies, summarizing the demographic data. Means and standard deviations were computed to identify the salient constructs perceived by dental students. Cronbach’s alpha (α) was calculated to assess the reliability of each construct. This reliability evaluation was conducted to verify the internal consistency of the construct items and to ascertain the accuracy of the measurement of the same constructs. Simple linear regression was employed to examine the association between each SFDP construct and the outcome “clinical teaching effectiveness”. To evaluate the predictive capability of the entire model, multiple linear regression was employed to analyze the combined impact of the SFDP constructs on clinical teaching effectiveness. An alpha level of 0.05 was established, and the Statistical Package for the Social Sciences (SPSS, Version 29.0) was utilized for all analyses. The responses to the open-ended questions were meticulously examined by the investigators to extract relevant and meaningful insights from the students. The SFDP constructs served as an analytical lens to identify noteworthy quotes. Relevant segments of text were coded and indexed according to the corresponding constructs. It is important to note that the aim of conducting this qualitative analysis was not to identify new themes, but rather to provide additional insights and a deeper understanding of the students’ perceptions.

## 3. Results

### 3.1. Demographics

A total of 332 responses were received, representing a 94% response rate. The survey respondents consisted of 57.5% females and 42.5% males. The median age of the respondents was 22 years. The majority of respondents, 87%, were predoctoral students, while 13% were graduate students.

### 3.2. Descriptive Statistics of the Proposed Model

Descriptive statistics, including means and standard deviations, were used to describe the model constructs (Table 1). The means for all of the SFDP constructs were above 4, ranging from 4.174 to 4.516. The feedback construct had the highest mean, while the construct for promoting self-directed learning had the lowest mean. The mean for clinical teaching effectiveness was 3.817, indicating lower student agreement with the clinical teaching received during the program.

Cronbach’s alpha was employed to assess the internal consistency of the model construct items and to determine the degree of correlation among them. The reliability scores ranged from 0.607 to 0.783 (Table 1).

### 3.3. Hypothesis Testing Findings

To examine the first seven hypotheses, simple linear regression analyses were conducted to ascertain whether significant relationships existed between each SFDP construct and the dependent variable “clinical teaching effectiveness”.

The results revealed a significant positive correlation between the SFDP constructs and clinical teaching effectiveness, thereby supporting all seven proposed hypotheses (Table 2). The evaluation construct exhibited the strongest correlation (r = 0.48, *p* < 0.001), accounting for 23.1% of the variance in clinical teaching effectiveness (F(1, 330) = 98.87, R^2^ = 0.231, *p* < 0.001). The second strongest correlation was observed for the construct related to communicating clinical requirements and goals (r = 0.42, *p* < 0.001), which explained 17.3% of the variance in clinical teaching effectiveness (F(1, 330) = 68.827, R^2^ = 0.173, *p* < 0.001). Conversely, the lowest correlation was associated with the construct of promoting understanding and retention (r = 0.33, *p* < 0.001), which accounted for 11% of the variance in clinical teaching effectiveness (F(1, 330) = 40.947, R^2^ = 0.110, *p* < 0.001) (Table 2).

To evaluate the overall model and test the eighth hypotheses, multiple linear regression was conducted to examine the collective influence of the SFDP constructs on predicting clinical teaching effectiveness. The results demonstrated that the SFDP model significantly predicted clinical teaching effectiveness (F(3, 328) = 1.495, R^2^ = 0.283, *p* < 0.001), thereby supporting the eighth hypothesis. The regression analysis indicated that the combined independent variables accounted for 28.3% of the variance in the clinical teaching effectiveness (see Table 2).

### 3.4. Open Ended Question Analysis

A total of 53 responses to the open-ended questions were received. The analysis of open-ended questions supported the SFDP constructs and provided deeper insights into students’ perceptions of the clinical teacher criteria they valued in clinical teaching. The students frequently mentioned the importance of providing a supportive learning climate, highlighting how clinical teachers could foster such an environment. For example, one student mentioned, “*Respect and understanding make a big difference in creating a positive learning environment. We need supervisors who are patient, empathetic, and supportive* (S.36)”. Additionally, in several responses, the students emphasized that clinical teachers should respect students in front of patients. One student stated that “*The clinical teachers should demonstrate respect towards me in the presence of patients, even if I am unable to answer a question, as I am still a student* (S.2)”. Another student added, “*Each clinical supervisor must respect me. If I did something wrong, he/she shouldn’t talk or raise his/her voice in front of patients* (S.17)”.

Students also highlighted the promotion of understanding and retention and their need for clear explanations of clinical procedures. For example, one student said “*I really appreciate a clinical teacher who explains clinical procedures well. It makes learning so much easier and more enjoyable when I can understand the procedure clearly* (S.29)”. Additionally, they underscored the importance of clear communication and interactive teaching methods in promoting effective clinical instruction. For instance, one student mentioned that “*A clinical teacher should be patient and ask questions to keep the discussion going. This makes learning more engaging and helps us understand things better. By encouraging open communication, supervisors can really help us grow and learn more effectively* (S.30)”.

Other key insights pertained to the evaluation and feedback constructs with students preferring consistent feedback and fair evaluation practices throughout the year. For example, one student highlighted that “*Some supervisors are lenient throughout the year, but on exam day, they start caring about every detail. Mistakes should be identified and addressed throughout the year, not just on the day of the exam* (S.35)”. Another student mentioned that “*The clinical teacher should let us know when we’re doing a good job or not. Positive or negative feedback is super important for us as students. It helps us see what we’re doing right and boosts our confidence* (S.38)”.

## 4. Discussion

This research aimed to identify key SFDP constructs related to dental students’ clinical teaching, with all seven constructs being effective predictors of the effectiveness of clinical teaching. The evaluation construct had the highest correlation, followed by the communication of clinical goals and the learning climate, underscoring the value of the SFDP constructs within the proposed model.

The evaluation construct was the strongest predictor of dental students’ clinical teaching effectiveness, explaining 23.1% of the variance. This significant correlation could be attributed to factors such as students expecting fair assessments using standardized criteria, which ensures consistency and clear expectations. Evaluating students’ ability to apply knowledge to specific patients’ needs was crucial for assessing critical thinking and problem-solving skills, essential for effective patient care. This finding is in line with previous research highlighting that evaluation is integral to the learning process and helps narrow the gap between actual and desired performance [21]. However, clinical assessments can be challenging due to the need to balance educational objectives with patient care responsibilities, leading to potential conflicts [22,23]. The variability in patient cases and the dynamic nature of clinical environments also complicate standardized assessments [22,23]. Students highlighted this challenge, emphasizing the need for clinical teachers to show them respect in front of patients. Despite this, clinical teachers should adopt a balanced approach to instructing students while ensuring patient safety and minimizing tension, especially during chairside interactions.

Interestingly, the communication of clinical goals construct was the second most important factor for the students. Explicit clinical requirements are invaluable as when students understand the expectations, they can focus on mastering specific skills and procedures. Awareness of expected competencies ensures that students are prepared for clinical practice. However, prior studies highlighted difficulties in attaining clinical practice goals due to a shortage of suitable patients and increased student enrollment [24,25]. This, combined with reduced teaching staff, has adversely affected staff-to-student ratios, potentially compromising education quality [24,25].

In this study, the learning climate was also significantly associated with clinical teaching effectiveness. Dental students perceived criteria such as being a good listener, showing respect, and demonstrating professionalism as key components in clinical teaching. These elements contribute to a positive learning environment essential for effective education. Previous studies support these findings. For instance, a study of 924 dental students showed that the learning climate positively influenced their basic psychological needs, such as autonomy, competence, and relatedness [26]. Similarly, Artim et al. reported a consensus among faculty members that proficient clinical instructors should exhibit clear communication skills, cultivate interpersonal relationships, and promote an atmosphere of mutual respect. Additionally, they should serve as exemplary role models, demonstrating emotional intelligence, compassion, trustworthiness, fairness, honesty, and supportiveness [27]. This suggests that a supportive learning environment can boost students’ motivation and enhance their overall educational experience. Furthermore, a qualitative study with nursing students found that clinical instructors who were motivated, engaged, and enthusiastic significantly encouraged students to immerse themselves more deeply in their studies [28].

The present study revealed significant correlations between the promotion of self-directed learning and feedback constructs with clinical teaching effectiveness in line with the literature. Lim et al. described effective feedback as an essential scaffold for self-directed learning, supporting the medical education process and fostering the development of students’ skills and knowledge [29]. Furthermore, these findings are consistent with Vygotsky’s theory of learning, known as the Zone of Proximal Development (ZPD). This theory, widely explored in educational research, encourages the development of models that prioritize the growth of learners’ cognitive abilities and personal development over simple rote memorization [30].

### 4.1. Limitations and Future Research Directions

The present study demonstrated that dental students’ perceptions of their clinical teaching were significantly influenced by all SFDP constructs. Although the model achieved significant results in predicting clinical teaching effectiveness, the explained variance was lower than anticipated, highlighting the need for further research to explore additional factors that might have impacted the effectiveness of clinical teaching. Factors inherent to the clinical environment might impact teaching and learning, such as patient availability, the complexity of clinical cases, and the necessity for real-time decision-making. Additionally, the expertise and knowledge of clinical teachers might play a crucial role since highly skilled instructors who can effectively communicate their knowledge tend to be more successful in facilitating student learning.

Moreover, although this study received a high response rate, it was conducted in a single dental school and there may be differences in dental faculties, pedagogical approaches, and clinical curricula between institutions. Future research should involve multiple dental schools to capture a broader range of teaching environments and practices. Comparative studies across different institutions could provide comprehensive insights into the factors influencing clinical teaching effectiveness.

### 4.2. Recommendations

Since standardized evaluation is instrumental in enhancing clinical teaching effectiveness, institutions should implement consistent and transparent evaluation criteria across all clinical courses. This entails developing standardized rubrics that clearly outline the expectations for the application of clinical knowledge and clinical skills [31]. By ensuring that all students are assessed according to uniform criteria, clinical teachers can provide more objective and fair evaluations, thereby helping students understand their strengths and areas for improvement. Additionally, regular training sessions for clinical educators on the use of these standardized evaluation tools can further enhance the reliability and validity of the assessments.

It is also recommended that clinical teachers should set clear clinical requirements and expected competencies at the beginning of each clinical rotation. This can be achieved through detailed syllabi, orientation sessions, and regular check-ins with students. Setting clear deadlines for clinical tasks and assessments can also help students manage their time effectively and stay on track with their learning objectives. Moreover, incorporating self-assessment opportunities allows students to reflect on their progress and identify areas where they need additional support, fostering a more proactive and self-directed approach to learning.

A positive learning climate is essential for effective clinical teaching, so clinical teachers should actively listen to students’ concerns, questions, and feedback [28]. This not only helps to address individual learning needs but also fosters a supportive and inclusive environment. By showing genuine interest in students’ perspectives, educators can build trust and encourage open communication, which is crucial for effective learning. Educators should treat all students with respect, regardless of their background or level of expertise by acknowledging their efforts, providing constructive feedback, and maintaining a professional attitude. A respectful environment promotes mutual respect among students and educators, enhancing collaboration and learning outcomes. Additionally, demonstrating respect towards patients sets a positive example for students, reinforcing the importance of professionalism in clinical practice.

Finally, fostering a supportive learning environment is essential for effective clinical teaching. Educators should prioritize creating a positive and inclusive atmosphere where students feel comfortable seeking guidance and feedback. This can be facilitated through regular one-on-one mentoring sessions in which teachers provide constructive feedback and encourage open communication. Additionally, promoting a culture of continuous improvement by encouraging students to set personal learning goals and providing opportunities for peer feedback can further enhance the learning experience.

## 5. Conclusions

In conclusion, this study emphasizes the significance of evaluation quality, the clear communication of clinical objectives, and the provision of supportive learning environments. Furthermore, supervisors’ respect for students promoted effective clinical education. Therefore, clinical teachers should carry out the following:

- Ensure high-quality evaluations to provide meaningful feedback to help students identify their strengths and areas for improvement.
- Clearly communicate clinical objectives to ensure that students understand what is expected of them, thereby reducing anxiety and increasing their confidence.
- And foster a supportive learning environment to encourage active participation and engagement, making the learning process more enjoyable and effective. Respectfulness not only enhances the learning experience but also models professional behavior that students can emulate in their future practice.

## Figures and Tables

**Table 1:** The proposed model constructs and its measuring scale items.

Constructs	Items	Mean (Std. Deviation)	Cronbach’s (α)
Learning climate	Good listener. Respectful. Encourages problem-solving. Demonstrates enthusiasm Professional/attentive to patients	4.384 (0.615)	0.783
Control of the session	Adheres to schedule timeframe. Punctuality. Sets agenda/guidelines.	4.253 (0.717)	0.655
Communication of clinical goals	Clear clinical requirements. Expected competence. Clear deadlines.	4.337 (0.639)	0.666
Promotion of understanding and retention	Structured explanation. Uses visual aids. Demonstrates multiple viewpoints.	4.277 (0.716)	0.706
Evaluation	Standardized evaluation. Clinical knowledge application. Clinical skills evaluation. Self-assessment.	4.185 (0.689)	0.754
Feedback	Corrective feedback. Explain right/wrong. Improvement suggestions.	4.516 (0.646)	0.781
Promotion of self-directed learning	Encourages further learning. Motivates independent learning. Assists with daily clinical problems.	4.174 (0.698)	0.607
Clinical teaching effectiveness	Effective clinical teaching Satisfactory clinical teaching Meets expectations	3.817 (0.836)	0.769

**Table 2:** Correlation coefficients between clinical teaching effectiveness and each SFDP construct.

Hypotheses	Constructs	r	R^2^	*p*	Hypothesis Results
H1	Learning climate	0.40	16%	<0.001	Supported
H2	Control the session	0.36	12.9%	<0.001	Supported
H3	Communication of clinical goals	0.42	17.3%	<0.001	Supported
H4	Promotion of understanding and retention	0.33	11%	<0.001	Supported
H5	Evaluation	0.48	23.1%	<0.001	Supported
H6	Feedback	0.38	14.4%	<0.001	Supported
H7	Promotion of self-directed learning	0.38	14.5%	<0.001	Supported
H8	Clinical teaching effectiveness	0.53	28.3%	<0.001	Supported

## Data Availability

The authors confirm that the data supporting the findings of this study are available upon reasonable request.

## Abbreviations

The following abbreviations are used in this manuscript:

SFDP	Stanford Faculty Development Program

## References

[1] Schönwetter D.J., Lavigne S., Mazurat R., Nazarko O. (2006). Students’ Perceptions of Effective Classroom and Clinical Teaching in Dental and Dental Hygiene Education. J. Dent. Educ..

[2] Wu J.H., Lin P.C., Lee K.T., Liu H.L., Lu P.Y., Lee C.Y. (2024). Situational simulation teaching effectively improves dental students’ non-operational clinical competency and objective structured clinical examination performance. BMC Med. Educ..

[3] Ismail L.M.N., Aboushady R.M.N., Eswi A. (2016). Clinical instructor’s behavior: Nursing student’s perception toward effective clinical instructor’s characteristics. J. Nurs. Educ. Pract..

[4] Niederriter J.E., Eyth D., Thoman J. (2017). Nursing students’ perceptions on characteristics of an effective clinical instructor. SAGE Open Nurs..

[5] Maart R., Gordon N. (2018). Dental clinical teachers’ perceptions of their teaching role. S. Afr. Dent. J..

[6] Treasure E., Durward C., Schwarz E. (2018). A Career in Dental Education. Career Paths in Oral Health.

[7] Sharka R. (2024). Factors associated with predoctoral and graduate dental students’ intention to care for elderly patients: A cross-sectional study. J. Dent. Educ..

[8] Shoaib L.A., Safii S.H., Naimie Z., Ahmad N.A., Sukumaran P., Yunus R.M. (2018). Dental students’ perceptions on the contribution and impact role of a clinical teacher. Eur. J. Dent. Educ..

[9] Khani H., Ahmady S., Sabet B., Namaki A., Zandi S., Niakan S. (2023). Teaching-learning in clinical education based on epistemological orientations: A multi-method study. PLoS ONE.

[10] Asadi M., Noorian S., Motefakker S., Heydari F., Shahsavari N., Senmar M. (2023). The state of clinical education and factors affecting effective clinical education: The point of view of nursing and midwifery students. BMC Med. Educ..

[11] Davis S., Duane B., Loxley A., Quigley D. (2022). The evaluation of an evidence-based model of feedback implemented on an undergraduate dental clinical learning environment. BMC Med. Educ..

[12] Johnsen D.C., Williams J.N., Baughman P.G., Roesch D.M., Feldman C.A. (2015). New Dental Accreditation Standard on Critical Thinking: A Call for Learning Models, Outcomes, Assessments. J. Dent. Educ..

[13] Kossioni A.E., Lyrakos G., Ntinalexi I., Varela R., Economu I. (2014). The development and validation of a questionnaire to measure the clinical learning environment for undergraduate dental students (DECLEI). Eur. J. Dent. Educ..

[14] Fluit C.R., Bolhuis S., Grol R., Laan R., Wensing M. (2010). Assessing the quality of clinical teachers: A systematic review of content and quality of questionnaires for assessing clinical teachers. J. Gen. Intern. Med..

[15] Jeong Y.N., Natto Z.S., Marks M.E., Karimbux N. (2020). Development and Implementation of a Clinical Dental Faculty Evaluation Instrument. J. Dent. Educ..

[16] Modha B. (2020). Experiential learning without prior vicarious learning: An insight from the primary dental care setting. Educ. Prim. Care.

[17] Stanford Faculty Development Center for Medical Teachers. https://med.stanford.edu/sfdc/clinical_teaching.html.

[18] Litzelman D.K., Stratos G.A., Marriott D.J., Skeff K.M. (1998). Factorial validation of a widely disseminated educational framework for evaluating clinical teachers. Acad. Med..

[19] Owolabi M.O. (2014). Development and Psychometric Characteristics of a New Domain of the Stanford Faculty Development Program Instrument. J. Contin. Educ. Health Prof..

[20] Mintz M., Southern D.A., Ghali W.A., Ma I.W.Y. (2015). Validation of the 25-Item Stanford Faculty Development Program Tool on Clinical Teaching Effectiveness. Teach. Learn. Med..

[21] Burgess A., van Diggele C., Roberts C., Mellis C. (2020). Feedback in the clinical setting. BMC Med. Educ..

[22] Fugill M. (2005). Teaching and learning in dental student clinical practice. Eur. J. Dent. Educ..

[23] Eriksen H.M., Bergdahl J., Bergdahl M. (2008). A patient-centred approach to teaching and learning in dental student clinical practice. Eur. J. Dent. Educ..

[24] McGleenon E.L., Morison S. (2021). Preparing dental students for independent practice: A scoping review of methods and trends in undergraduate clinical skills teaching in the UK and Ireland. Br. Dent. J..

[25] Sabato E., Doubleday A.F., Lee C.T., Correa L.P., Huja S., Crain G. (2023). Recommendations for remaining agile in the face of a dental faculty workforce shortage. J. Dent. Educ..

[26] Orsini C., Binnie V., Wilson S., Villegas M.J. (2018). Learning climate and feedback as predictors of dental students’ self-determined motivation: The mediating role of basic psychological needs satisfaction. Eur. J. Dent. Educ..

[27] Artim D.E., Smallidge D., Boyd L.D., August J.N., Vineyard J. (2020). Attributes of effective clinical teachers in dental hygiene education. J. Dent. Educ..

[28] Soroush A., Andaieshgar B., Vahdat A., Khatony A. (2021). The characteristics of an effective clinical instructor from the perspective of nursing students: A qualitative descriptive study in Iran. BMC Nurs..

[29] Lim Y.S., Willey J.M., Bangeranye C. (2024). An Exploration of Cognitive Diagnosis in Medical Education: Constructing Comprehensive Feedback for Enhanced Student Learning. Med. Sci. Educ..

[30] Margolis A.A. (2020). Zone of Proximal Development, Scaffolding and Teaching Practice. Cult.-Hist. Psychol..

[31] Dreiling K., Montano D., Poinstingl H., Müller T., Schiekirka-Schwake S., Anders S., von Steinbüchel N., Raupach T. (2017). Evaluation in undergraduate medical education: Conceptualizing and validating a novel questionnaire for assessing the quality of bedside teaching. Med. Teach..

